# Use of miniplates as a method for orthodontic anchorage: a case report

**DOI:** 10.1590/2177-6709.21.5.095-102.oar

**Published:** 2016

**Authors:** Fernando Gianzanti Peres, Luis Eduardo Marques Padovan, Leandro Eduardo Kluppel, Gustavo Calvalcanti Albuquerque, Paulo Cesar Ulson de Souza, Marcela Claudino

**Affiliations:** 1 MSc in Implantology, Instituto Latino Americano de Pesquisa e Ensino Odontológico (ILAPEO), Curitiba, Paraná, Brazil.; 2 Professor of Implantology, Instituto Latino Americano de Pesquisa e Ensino Odontológico (ILAPEO), Curitiba, Paraná, Brazil.; 3 Professor, Universidade do Estado do Amazonas (UEA), Department of Oral and Maxillofacial Surgery, Manaus, Amazonas, Brazil.; 4 DDS, Instituto Odontológico de Cirurgia e Prótese (IOCP), Bauru, São Paulo, Brazil.; 5 Professor, Universidade Estadual de Ponta Grossa (UEPG), Graduation Course, Ponta Grossa, Paraná, Brazil.

**Keywords:** Tooth movement. Orthodontic anchorage procedures. Molar. Tooth, Impacted.

## Abstract

**Introduction:**

Temporary anchorage devices (TADs) have been developed to be used as direct adjuncts in orthodontic treatment and have facilitated treatment of more complex orthodontic cases, including patients with dental impaction.

**Objectives:**

This clinical case reports the applicability of TADs in the orthodontic treatment of a patient with impacted mandibular second molars. Surgical and orthodontic procedures related to the use of miniplates were also discussed in this study.

**Conclusions:**

The use of temporary anchorage devices, such as miniplates, can be suggested as an alternative to treat patients with impacted mandibular second molars.

## RESUMO

**Introdução:**

os dispositivos de ancoragem temporária vêm sendo desenvolvidos para uso como coadjuvantes no tratamento ortodôntico. Esses dispositivos facilitam o tratamento ortodôntico de casos mais complexos, incluindo pacientes com dentes impactados.

**Objetivos:**

o presente relato de caso reporta a aplicabilidade dos dispositivos de ancoragem temporária no tratamento ortodôntico de um paciente com segundos molares inferiores impactados. Os procedimentos cirúrgicos e ortodônticos relacionados ao uso das miniplacas também são discutidos nesse estudo.

**Conclusões:**

o uso de dispositivos de ancoragem temporária, tais como as miniplacas, pode ser sugerido como uma alternativa no tratamento de pacientes com segundos molares inferiores impactados.

## INTRODUCTION

Orthodontic anchorage has been considered relevant in providing satisfactory clinical results. Initially, in conventional treatment, headgear and intraoral elastics are used to improve anchorage. However, these devices require patient's compliance, which can significantly influence treatment.^1^ In this context, temporary anchorage devices (TADs) have been developed to be used as direct adjuncts in orthodontic treatment, including conventional implants, titanium miniscrews, and bone miniplates. These devices have demonstrated favorable clinical results and have facilitated treatment of more complex orthodontic cases.^2^

Complex cases include patients with dental impaction. Conceptually, impacted teeth are the result of eruption impairment due to the presence of physical barriers in the tooth eruption pathway or altered position of the tooth germ. In a general context, tooth impaction affects about 20% of the population. However, when specific dental groups are assessed, prevalence rates demonstrate significant differences. For example, reduced rates of 0.08% and 0.01% are reported for impaction of mandibular first molars and second molars, respectively.^3^ Despite low prevalence, orthodontic treatment of these cases has a higher degree of complexity. Treatment options depend on the degree of tooth tipping, the position of third molars and the desired type of motion. Moreover, this treatment can be carried out by means of surgical or orthodontic procedures.^4^

As regards surgical alternatives, surgical repositioning is proposed as a therapeutic method for the treatment of impacted teeth. Although this procedure provides an immediate solution, complications, such as endodontic and periodontal impairment, have been reported.^5^ ^,^ ^6^ Orthodontic movement is also indicated for uprighting impacted molars achieved by means of fixed or removable appliances.^7^ ^,^ ^8^ ^,^ ^9^ In this context, the establishment of a distal force component is difficult because of the presence of impacted third molars, mainly in adolescent patients.^10^ ^,^ ^11^ In these cases, the applicability of skeletal anchorage optimizes orthodontic treatment and has advantages, such as decreased overall treatment time and effective anchorage with reduced aesthetic discomfort, when compared to extraoral anchorage. Occasionally, it allows orthodontic treatment previously thought to be impossible without surgery to be carried out. These devices also minimize the need for patient's compliance.^1^ ^,^ ^12^ However, disadvantages, such as surgical procedures for placement and removal of devices, as well as inflammation of surrounding soft tissues, have been reported.^13^

Based on the relevance of the use of TADs and the higher degree of complexity implied in treating surrounding patients with impacted mandibular second molars, the aim of this study was to present a case report regarding the applicability of temporary anchorage devices (TADs) in the orthodontic treatment of a patient with impacted mandibular second molars. 

## CASE REPORT

**Figure 1:**
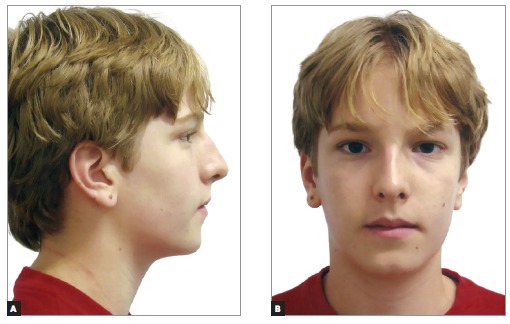
Initial facial aspect: A) lateral and B) frontal.

**Figure 2:**
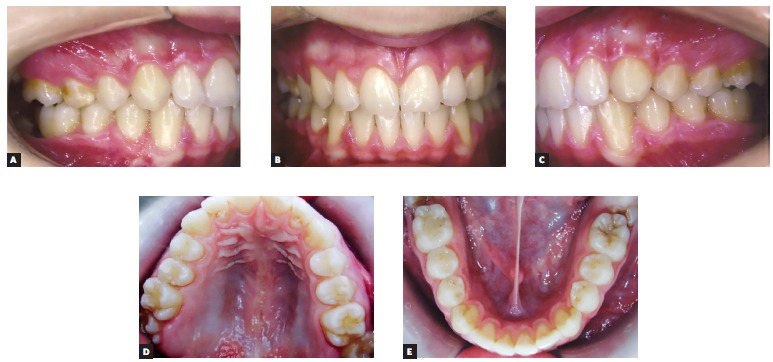
Initial clinical aspect: A) intraoral right side, B) intraoral frontal aspect, C) intraoral left side, D) occlusal maxillary aspect, and E) occlusal mandibular aspect.

**Figure 3:**
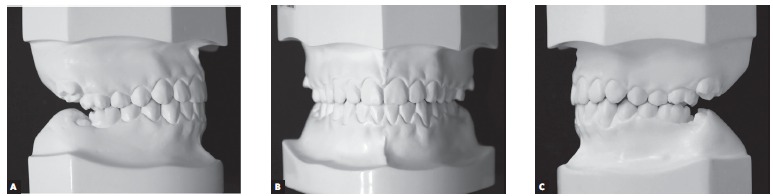
Initial dental casts: A) Right side aspect, B) frontal aspect, C) left side aspect.

**Figure 4:**
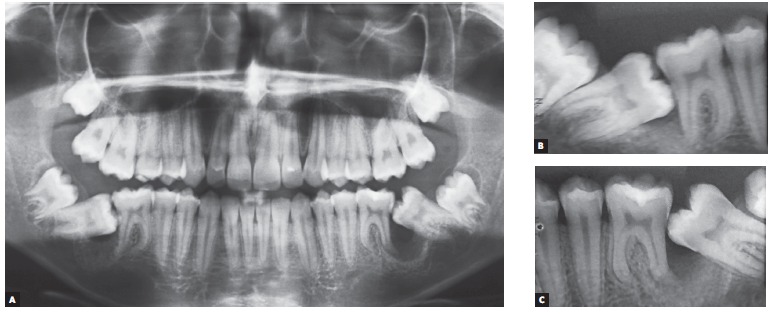
Initial radiographic aspect: panoramic radiograph (A) and periapical radiographs of teeth #46 and #47 (B), and #36 and #37 (C).

A 14 year-old boy with permanent dentition was referred for treatment after complaining of a need to correct dental position. Evaluation revealed a balanced facial growth pattern, convex profile and passive lip seal. Furthermore, the following were identified: dental alignment, Class I canine relationship and coinciding midlines (Figs 1, 2 and 3). Mandibular second molars were severely mesially tipped and semi-erupted; impacted third molars were also observed (Fig 4). Radiographic examination also revealed the presence of inflammatory cysts in the region of mandibular left second and third molars and in the region of maxillary left second and third molars. Thus, treatment planning was based on cysts enucleation and uprighting of mandibular second molars (#37 and #47) achieved by means of miniplates.

Treatment was initiated with enucleation of cysts. Subsequently, a miniplate for orthodontic anchorage was installed with an incision in the molar and premolar regions on both sides, and with raising of a mucoperiostal flap in the zygomatic bone region.

The miniplate (Neoortho, Curitiba, Brazil), 22.16 mm in lenght, 1 mm in thickness and 13.5-mm wide, was adapted on both sides of the maxilla. Fixation was performed with three screws (1.5 mm in diameter and 5 mm in length) on each side. After fixation and manual torque, the suture was performed with Monocryl 4-0 (Ethicon, São Paulo, Brazil). Mechanics was initiated after 15 days with orthodontic traction of mandibular second molars (#37 and #47).

**Figure 5:**
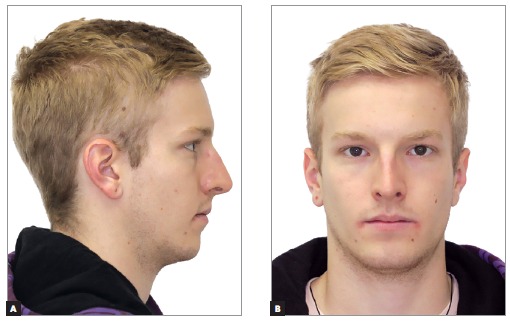
Final facial aspect. A) lateral and B) frontal.

**Figure 6:**
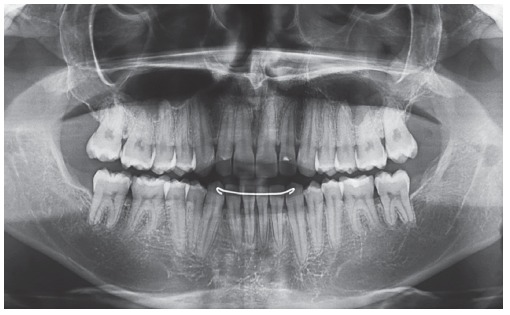
Final radiographic aspect.

**Figure 7:**
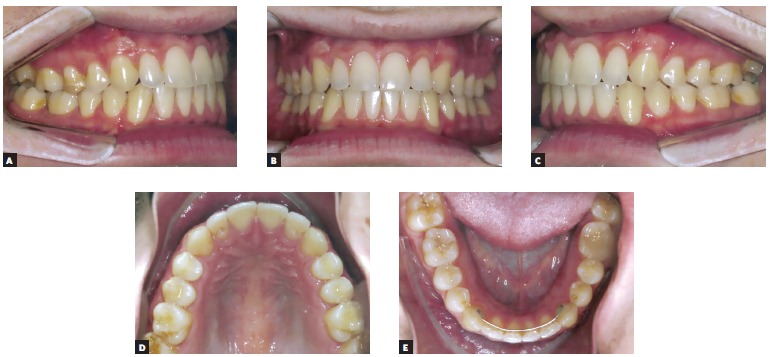
Final clinical aspect: A) intraoral right side, (B) intraoral frontal, C) intraoral left side, D) occlusal mandibular aspect, and E) occlusal maxillary aspect.

**Figure 8:**
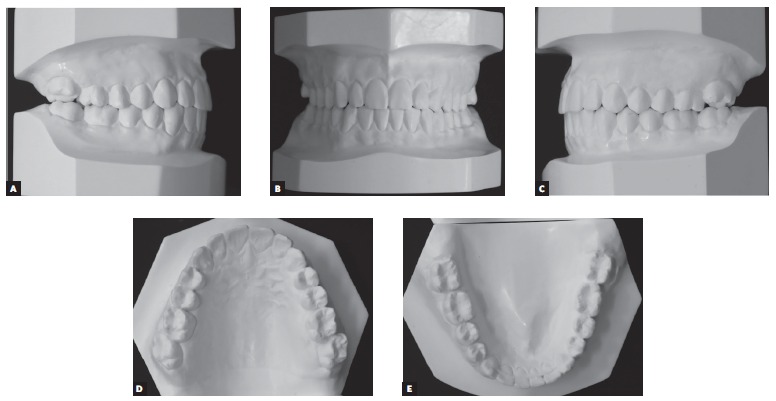
Final dental casts. (A) Right side aspect, (B) frontal aspect, (C) left side aspect, and (D, E) occlusal aspect.

**Figure 9:**
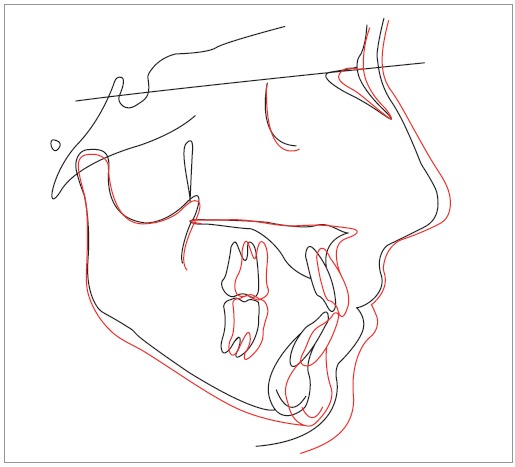
Superimposition of initial (black) and final (red) cephalometric tracings.

**Figure 10:**
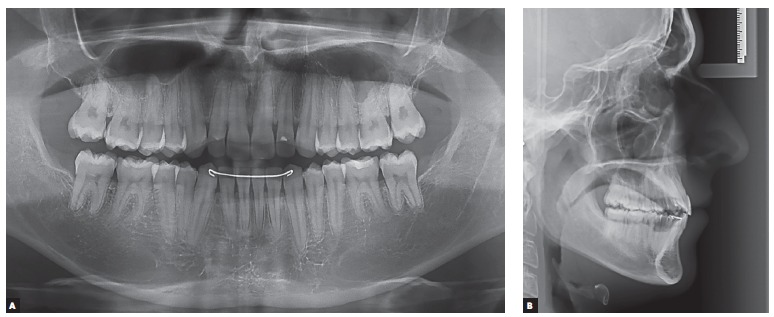
Radiographic follow-up two years after orthodontic treatment completion.

Buttons were placed on the buccal surface of mandibular second molars, followed by placement of 1/4 intermaxillary elastics with a force of 150 g (mouth closed) and approximately 200 g (mouth open). After five months, orthodontic miniplates were removed, since second molars were in proper position for uprighting with cantilevers. After uprighting, mandibular third molars were extracted, totaling 37 months of treatment (Figs 5, 6, 7, 8, and 9). After orthodontic treatment completion, the patient was followed-up for two years (Fig 10).

## DISCUSSION

The case described in this study demonstrates a relatively unusual event, since the most common dental impaction is unilateral.^14^ In this case, bilateral impaction was observed in the jaw in a male patient. Regardless of the treatment approach employed for impacted teeth, it is important that treatment be performed as soon as possible due to contact with adjacent teeth, which can result in root resorption, caries and periodontal diseases.^15^

For molar uprighting, several options of mechanical approaches are described, namely: cantilevers, tip-back spring, NiTi wire, among others.^7^ ^,^ ^8^ ^,^ ^16^ In the present case, the first approach employed for molar uprighting was the use of orthodontic elastics supported on miniplates in order to increase molar exposure in the oral cavity. As soon as possible, a cantilever was used to finish the movement. The cantilever is a segment of wire in which one end in inserted in a tube or bracket and the other end has just a point of contact. It was chosen in this case because, as a statistically determined system,^17^ ^,^ ^18^ it generates a predictable force system.^16^ ^,^ ^18^ ^,^ ^19^

The clinical results of the case reported herein demonstrate that the use of miniplates as anchorage devices was an efficient strategy for uprighting impacted second molars. The introduction of miniplate anchorage in Orthodontics was a great progress, since it minimizes the need for patient's compliance^7^ and allows for a more predictable orthodontic movement.^20^ ^-^ ^23^ Furthermore, the use of these devices enables the application of force from the distal side of the impacted molar. This application of force generates a counterclockwise moment, which allows movement control, thus promoting rapid disimpaction and crown distalization.^22^ ^,^ ^24^ Despite yielding good treatment results, miniplates are not yet widely used, possibly due to the requirement of surgical procedures with a certain degree of complexity, as well as the need for subsequent orthodontic treatment.

In fact, the majority of case reports describe the use of screws or microscrews.^25^ ^-^ ^28^ However, in the present case, the use of miniplates was a precise, safe and simple method for skeletal anchorage. Moreover, it does not require complex movements and involvement of several teeth in the process.^27^ Nevertheless, disadvantages over conventional devices are reported, including the need for surgical procedures, high cost, difficult cleaning, risk of infection and discomfort during the first days due to the size of the device^28^. Thus, the use of these devices may be indicated for treatment of specific cases.

## CONCLUSIONS

This case report demonstrates the applicability of miniplates for uprighting impacted second molars. Thus, the use of these devices may be used as an alternative to treat patients with impacted mandibular second molars. 
